# Feeding the hospitalized large‐for‐gestational‐age infant: Clinical dilemmas and the need for growth standards

**DOI:** 10.1002/ncp.70114

**Published:** 2026-03-11

**Authors:** Nichole R. Cortez, Holly R. Hull, Danielle N. Christifano, Susan E. Carlson, Praveen S. Goday

**Affiliations:** ^1^ Clinical Nutrition, Saint Luke's Health System Boise Idaho USA; ^2^ Nutrition and Dietetics, University of Kansas Medical Center Kansas City Missouri USA; ^3^ Department of Obstetrics and Gynecology University of Kansas Medical Center Kansas City Missouri USA; ^4^ Pediatric Gastroenterology and Nutrition, Nationwide Children's Hospital Columbus Ohio USA

**Keywords:** enteral nutrition, metabolic diseases, neonates, nutrition assessment, obesity

## Abstract

Infants born large‐for‐gestational‐age (LGA) are increasingly common because of the rising rates of maternal obesity and diabetes. Some infants born LGA require hospitalization and nutrition support, yet no specific growth standards exist to guide nutrition management. Feeding practices based on weight‐based guidelines for infants born at appropriate weight for gestational age may unintentionally promote overfeeding and increase long‐term obesity risk. This Clinical Dilemma explores the challenges of feeding hospitalized infants born LGA and highlights the urgent need for research to develop evidence‐based growth standards for this at‐risk population.

## INTRODUCTION

Childhood obesity affects 1 in 5 children in the United States, and projections suggest up to 50% of children may be overweight or obese by 2033.^1^ Obesity‐related conditions begin early, and birth weight plays a critical role. Approximately 10% of US infants are born large‐for‐gestational‐age (LGA, >90th percentile weight for gestational age), and 8% are macrosomic (>4 kg).^2^ Women with obesity are more likely to give birth to a large baby, with approximately 22.3% of women who are obese giving birth to a macrosomic infant compared with 7.7% of women who are normal weight.^3^ Additionally, infants of mothers with type 1, type 2, or gestational diabetes (IDMs) are also more likely to be LGA.^4^ Children who are overweight or obese are at a higher risk of comorbidities, including metabolic syndrome.^5^ Furthermore, children who are overweight or obese are more likely to remain so into adulthood. This predisposes them to a higher risk of developing chronic conditions such as diabetes and heart disease.^4^

Approximately 9% of infants born LGA require admission to the neonatal intensive care unit (NICU),^6^ where they may receive nutrition support that is necessary for their medical care. The goal of this narrative is to review feeding strategies for infants born LGA in the NICU and to justify the need to develop feeding guidelines and growth standards that are specific to the hospitalized infant born LGA to achieve an appropriate weight for age and, perhaps, decrease long‐term risks.

### Clinical dilemma

The central dilemma is that although infants born LGA often require nutrition support, no evidence‐based feeding guidelines exist for this subgroup. Current NICU feeding practices are designed to promote weight gain and growth. For infants born at term, they should grow per World Health Organization (WHO) growth standards, which reflect growth patterns established in typical, breastfed infants.^7^ This approach does not account for the altered body composition of infants born LGA, particularly the IDMs who often present with higher mass but not necessarily higher lean mass or energy needs.^8^ Clinicians often determine initial feeding volumes based on birth weight, which may inadvertently lead to overfeeding, prolonged hospitalization, or even programming of unhealthy growth trajectories.^9^ Given the lack of research regarding appropriate weight gain in the in these infants, there is concern that providing less than optimal nutrition support may risk poor outcomes such as poor weight gain, hypoglycemia, and disrupted neurodevelopment.^10^

#### LGA births and growth

Infants born LGA who grow in a hyperglycemic and hyperinsulinemic environment are born with greater weight and adiposity as compared with an infant of the same gestational age but born at an appropriate weight for gestational age (AGA).^4^ It is hypothesized that nutrition and body composition changes in the early weeks of life play a critical role in long‐term metabolic development.^11^ Some infants have a large body size stemming from genetics and normal intrauterine growth; understanding which infants born LGA are most vulnerable is critical in assessing this dilemma.

Not all infants born LGA are the same, with some more likely to be admitted to the NICU than others. Infants born LGA who were a smaller size at midgestation with “catch‐up” growth to term associated with increased abdominal circumference had the highest risk of macrosomia and admission to the NICU.^12^ In contrast, Bommarito et al noted that infants who were consistently large throughout gestation had a low risk of adverse birth events, including NICU admission.^12^ The former was more likely to have a mother with abnormal blood glucose and pregestational diabetes. It is the IDMs who are more at risk for instability that requires tube feeding and who are most at risk for long‐term adverse health outcomes.^5^, ^13^, ^14^

For infants who are born LGA and allowed to feed ad libitum, the rate of weight gain may naturally slow.^15^, ^16^, ^17^ The process of slowed weight gain in infancy has been coined “catch‐down growth.” A healthy infant born LGA at term may experience a catch‐down growth of up to −0.67 weight‐for‐age *z* score (WAZ) to achieve a normal weight for age or body proportionality by 6 months without adverse long‐term metabolic consequences.^18^ However, too rapid a decline in the rate of weight gain can result in failure‐to‐thrive and threaten normal development, with children in one study whose weights dropped below the 50th percentile also having longer‐term adverse outcomes, such as growth restriction and lower IQ scores at 7 years old.^18^ Given the above, the most appropriate catch‐down growth has yet to be firmly established; however, there is evidence that for some infants born LGA, achieving a more normal weight for length can reduce the risk of metabolic problems such as obesity and high blood pressure in childhood.^19^ Infants born LGA who remain obese into adolescence and adulthood are more likely to suffer from noncommunicable chronic disease, including metabolic syndrome.^5^, ^13^, ^20^

#### Feeding infants born LGA

IDMs who are born LGA experience a higher rate of birth complications that require hospitalization^14^ and disrupt normal feeding patterns and breastfeeding initiation. These include complications such as birth injury, respiratory insufficiency, and hypoglycemia requiring enteral tube feeding and/or administration of intravenous nutrition.^21^ Some also experience poor oral feeding due to low muscle tone and low feeding interest and take longer to achieve full feeds than their appropriately grown counterparts of the same gestation.^22^, ^23^ In the setting of poor oral intake and low interest, the LGA IDM admitted to the NICU will often receive enteral tube feeding nutrition support. Underfeeding may exacerbate poor oral intake and hypoglycemia, but overfeeding can also impact oral feeding and may increase body fatness. Based on what is known about early excess weight gain in relation to long‐term risk of overweight and obesity,^24^ it is not desirable to overfeed infants born LGA in the NICU.

#### Enteral nutrition

Multiple medical and developmental factors contribute to poor feeding in IDMs, making early enteral support common in this group. Poor feeding is common in infants born LGA, and when combined with increased risk of hypoglycemia from the IDM, this leads to an increased likelihood of the need for transitional enteral feedings. In the absence of adequate oral intake from the infant, however, the provider must determine the enteral feeding volumes.

Although overfeeding is a major concern, underfeeding also carries significant risks, particularly for IDMs born LGA who are vulnerable to hypoglycemia and often require specialized feeding strategies. Because tube feeding calculations are often based solely on weight, they may not account for the altered body composition of LGA IDMs, increasing the risk of overfeeding. Often, a dextrose infusion is required to normalize blood glucose levels. Enteral feeds can aid in sustaining blood glucose and reduce the need for dextrose infusions. In a poorly eating infant, enteral feeds provide a reliable supply of nutrition to maintain normoglycemia. Although hypoglycemia is typically transient, the enteral feeding tube may remain in place given the risk of refractory hypoglycemia in an infant with minimal oral intake or to supplement perceived poor or slow voluntary feeding.^23^, ^25^

#### Ad libitum oral feeding

Ad libitum feeding may serve as the best reference for energy needs in infants born LGA. Understanding normal intake from ad libitum feeding of healthy infants born LGA may be useful in estimating needs for infants who require enteral feeding. If allowed to feed ad libitum, infants born LGA may achieve a more proportional body composition without intervention and may experience a decline in weight‐for‐age trajectory.^24^ Promoting good breastfeeding practices may also protect against excessive weight gain.^17^ Research examining overweight and obese mothers and those with excessive gestational weight gain suggests that infants who are breastfed longer have a reduced weight‐for‐height and improved height‐for‐age score in childhood.^26^, ^27^ Breastfeeding has the advantage in that the mother is more likely to feed based on cues, whereas bottle‐feeding may encourage the caretaker to override ad libitum feeding by insisting the infant finish what is in the bottle.

#### Proposed growth trajectories for LGA newborns requiring nutrition support

There are no guidelines for weight gain for infants born LGA. Ideally, they would be breastfed and grow at predetermined weight velocities. However, some infants born LGA require medical and concomitant nutrition intervention in the form of enteral nutrition or, rarely, parenteral nutrition.

**Figure 1:**
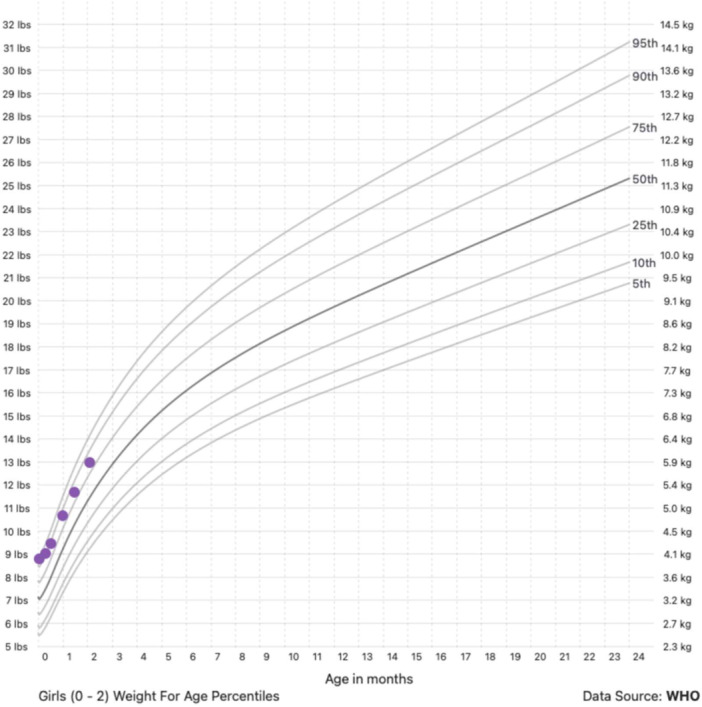
Example growth chart of a 4 KG LGA female infant following 50th percentile weight gain through 2 months of age.

Using the WHO weight velocity charts, we simulated weight gains along the 5th, 10th, 25th, and 50th percentiles for girls born LGA with birth weights of 3.85, 4, 4.25, and 4.5 kg and boys born LGA with birth weights of 4, 4.25, and 4.5 kg (Tables 1 and 2). Tables 1 and 2 illustrate that boys and girls with a ≤4‐kg birth weight can achieve a WAZ of +1 at 2 months of age by gaining weight at the 50th percentile, which could be achieved by targeting a weight gain in grams per day for the 50th percentile shown in Table 3. On the other hand, infants born with a >4‐kg birth weight could be encouraged to growth at rates less than the 50th percentile to achieve the same WAZ at 2 months of age. For example, a girl born at 4.5 kg could achieve a WAZ near +1 at 2 months (Tables 1 and 2) by gaining weight at the 25th percentile. Figure 1 shows an example of an LGA infant following the 50th percentile of weight gain. Girls born at 4.5 kg and boys born at 4.25 and 4.5 kg would gain too much if they gained weight at the 50th percentile, but they would be too restricted with weight gain at the 25th percentile, so some intermediate weight gain would need to be the goal.

**Table 1:** Simulated weight change in girls born large for gestational age based on the World Health Organization weight velocity growth charts.

	Birth weight															
	3.85 kg				4 kg				4.25 kg				4.5 kg			
	Weight change (in grams) at the				Weight change (in grams) at the				Weight change (in grams) at the				Weight change (in grams) at the			
	5th%ile	10th%ile	25th%ile	50th%ile	5th%ile	10th%ile	25th%ile	50th%ile	5th%ile	10th%ile	25th%ile	50th%ile	5th%ile	10th%ile	25th%ile	50th%ile
Day of life																
7	−250	−150	0	100	−250	−150	0	100	−200	−100	0	150	−200	−100	0	150
14	−100	0	100	200	−100	0	100	200	0	50	100	200	0	50	100	200
28	250	300	450	550	250	300	450	550	200	300	450	600	200	300	450	600
42	200	295	325	457	200	295	325	457	300	300	375	525	300	300	375	525
60	250	350	408	585	250	350	408	585	150	155	334	550	150	155	334	550
Weight at 60 days	4200	4645	5133	5742	4350	4795	5283	5892	4700	4955	5509	6275	4950	5205	5759	6525
*z* Score at 60 days	−1.52	−0.76	0.01	0.87	−1.26	−0.52	0.23	1.08	−0.67	−0.26	0.55	1.57	−0.27	0.11	0.9	1.87

*Note*: Term female infants are considered large for gestational age at a 3.85‐kg birth weight, whereas male infants are considered so at a 4‐kg birth weight.

Abbreviation: %ile, percentile.

**Table 2:** Simulated weight change in boys born large for gestational age based on the World Health Organization weight velocity growth charts.

	Birth weight											
	4 kg				4.25 kg				4.5 kg			
	Weight change (in grams) at the				Weight change (in grams) at the				Weight change (in grams) at the			
	5th%ile	10th%ile	25th%ile	50th%ile	5th%ile	10th%ile	25th%ile	50th%ile	5th%ile	10th%ile	25th%ile	50th%ile
Day of life												
7	−300	−250	0	150	−250	−250	−50	50	−250	−250	−50	50
14	−50	0	100	250	−100	50	150	275	−100	50	150	275
28	350	400	500	700	400	400	550	725	400	400	550	725
42	300	350	450	550	300	300	450	548	300	300	450	548
60	350	400	500	650	217	300	400	611	217	300	400	611
Weight at 60 days	4650	4900	5550	6300	4817	5050	5750	6459	5067	5300	6000	6709
*z* Score at 60 days	−1.43	−1.02	−0.03	1.01	−1.15	−0.78	0.26	1.22	−0.75	−0.4	0.61	1.53

*Note*: Term female infants are considered large for gestational age at a 3.85‐kg birth weight, whereas male infants are considered so at a 4‐kg birth weight.

Abbreviation: %ile, percentile.

**Table 3:** Weight gain in grams (g) per day based on the World Health Organization weight velocity chart.

	Girls				Boys			
	Birth weight ≤4 kg		Birth weight >4 kg		Birth weight ≤4 kg		Birth weight >4 kg	
	Weight gain, g/day		Weight gain, g/day		Weight gain, g/day		Weight gain, g/day	
Days of life	25th%ile	50th%ile	25th%ile	50th%ile	25th%ile	50th%ile	25th%ile	50th%ile
0–7	0	14	0	21	0	21	−7	7
7–14	14	29	14	29	14	31	25	36
14–28	32	42	31	44	36	50	37	50
28–42	25	32	26	38	33	41	31	40
42–60	23	32	20	29	26	34	23	34

*Note*: Children of both sexes born ≤4 kg should gain weight at the 50th percentile to achieve a weight‐for‐age z score of approximately +1 at 2 months of age. Children of both sexes born >4 kg should gain weight between the 25–50th percentile to achieve a weight‐for‐age z score of approximately +1 at 2 months of age.

Abbreviation: %ile, percentile.

Children of both sexes born ≤4 kg should gain weight at the 50th percentile to achieve a weight‐for‐age z score of approximately +1 at 2 months of age while children with birth weights >4 kg should gain weight between the 25–50th percentile to achieve a weight‐for‐age z score of approximately +1 at 2 months of age. Table 3 can be used to figure out the ‘expected’ weight gain at various timepoints in the first two months to enable these children to achieve appropriate catch‐down growth.

Figure 1 illustrates the WAZ trend of a term female infant born at 4 kg who follows the 50th percentile of weight gain through 2 months of age. Children at or below a 4.25‐kg birth weight can likely grow along the 50th percentile and still achieve this WAZ. For children closer to a 4.5‐kg birth weight, weight trajectories between the 25th and 50th percentile may help them achieve this WAZ.

These recommendations should be used with caution. The original WHO growth charts were created using children whose mothers had no significant medical history. These should likely not be used in premature infants, and neither should they be used in children who are well above 4.5 kg. Even in children between 3.85 and 4.5 kg, these should be used as rough guidelines, and careful clinical assessment of children should guide care.

## CONCLUSION

The current challenge in feeding hospitalized LGA newborns lies in the lack of published guidelines on healthy weight gain following normal postnatal weight loss. The increasing prevalence of infants born LGA, especially those born to mothers with obesity and diabetes, highlights the urgent need for evidence‐based nutrition guidance in early life.^28^, ^29^, ^30^ This, along with other key points highlighted in this paper, is summarized in Table 4.

**Table 4:** Summary of key practice points.

Practice focus	Key point
Limitations of current guidelines	Standard NICU feeding and growth guidelines are designed for infants born AGA and may not be appropriate for hospitalized infants born LGA, particularly those born to mothers with diabetes.
Weight‐based feeding	Reliance on weight alone to determine feeding volumes may overestimate energy needs and increase the risk of overfeeding in infants born LGA.
Risk balance	Nutrition management must balance preventing hypoglycemia and supporting feeding progression with avoiding excessive weight gain and adiposity.
Growth trajectory	Modest catch‐down growth in early infancy may be physiologic and metabolically beneficial for some infants born LGA. Following the 25th–50th percentile of weight gain per WHO weight gain trends may be a safe way to achieve this catch‐down growth.
Feeding strategy	When infants are medically stable, cue‐based, ad libitum oral feeding (especially breastfeeding) may best reflect physiologic energy needs.
Individualized care and research need	Until LGA‐specific standards exist, feeding decisions should be individualized, underscoring the urgent need for evidence‐based growth and nutrition guidelines for this population.

Abbreviations: AGA, appropriate for gestational age; LGA, large for gestational age; NICU, neonatal intensive care unit; WHO, World Health Organization.

Future research should distinguish among subgroups of infants born LGA to clarify which patterns of growth predict healthier metabolic and cognitive outcomes. We propose three research priorities that will help clinicians better understand the needs of this growing population: (1) defining normal intake for healthy infants born LGA; (2) determining healthy catch‐down growth that supports optimal neurodevelopment; and (3) developing unique growth standards for the population born LGA.

Defining normative intake ranges for infants born LGA is essential to ensure that feeding practices are both safe and effective. One means of obtaining such knowledge might be to observe normal intake in infants born LGA not admitted to the NICU who also achieve some catch‐down growth. This may be challenging in breastfed infants. Investigating the safety and feasibility of promoting catch‐down growth represents a second research priority.

In healthy term and late‐preterm infants who are born LGA, are fed ad libitum, and do experience catch‐down growth, the impact of slowed weight gain on neurodevelopment is also not yet fully understood. Intentional modulation of growth in the NICU requires careful evaluation to ensure that it does not compromise neurodevelopment or other aspects of health. Clinicians must balance the potential benefits of slowing weight gain against the risks of undernutrition during a critical developmental window. Much as described above, observing healthy infants born LGA who both do and do not experience catch‐down growth and conducting standardized development testing such as the Bayley scales or psychomotor index test over time can provide insights into the effects of different growth patterns. Once the safety of different growth trends and normative intakes are established, unique growth standards can be developed.

Evidence‐based growth standards for infants born LGA should aim to balance immediate nutrition adequacy with the promotion of long‐term metabolic health. Ideally, such standards would be derived from longitudinal data linking early growth trajectories with health outcomes across childhood and adolescence, as described above. The weight gain standards that we propose in this paper are derived from WHO growth charts, which, again, were created using infants born AGA at term. Studies that incorporate additional markers such as body composition and metabolic indicators and assess developmental milestones would strengthen the evidence and should be sought out in developing growth recommendations. Establishing such growth standards would empower clinicians to provide care that reduces overfeeding and protects against long‐term health risks.

The growing number of infants born LGA presents a critical challenge that requires targeted research and clinical innovation. Advancing knowledge in this area may be vital in reducing the burden of obesity and metabolic disease while ensuring optimal growth and neurodevelopment for infants born LGA.

## AUTHOR CONTRIBUTIONS

Nichole R. Cortez and Susan E. Carlson equally contributed to the conception and design of the research. Nichole R. Cortez, Susan E. Carlson, Holly R. Hull, and Danielle N. Christifano contributed to the design of the research. Nichole R. Cortez contributed to the acquisition and analysis of the data. Susan E. Carlson, Holly R. Hull, and Danielle N. Christifano contributed to the interpretation of the data; all authors drafted the article. All authors critically revised the article, agree to be fully accountable for ensuring the integrity and accuracy of the work, and read and approved the final article.

## CONFLICT OF INTEREST STATEMENT

None declared.
